# Any level of acute kidney injury may be associated with mortality in critically ill patients

**DOI:** 10.1186/cc9523

**Published:** 2011-03-11

**Authors:** AP Rucks, AF Meregalli, DA Becker, JM Andrade, G Friedman

**Affiliations:** 1 Complexo Hospitalar Santa Casa, Porto Alegre, Brazil

## Introduction

Acute kidney injury (AKI) is a common condition in critically ill patients [1]. It is an independent risk factor for in-hospital mortality in this population [2]. The goal of this research is to classify critically ill patients within RIFLE criteria [3] and assess its impact on 30-day in-hospital mortality.

## Methods

From September 2009 to July 2010, all patients admitted to two ICUs of Santa Casa Hospital were included in this study. Age, gender, SOFA and APACHE scores, origin, serum creatinine, whether they were clinical or surgical, and outcome were noted. Then patients were classified as 'no AKI', 'risk', 'injury', or 'failure' according to RIFLE criteria. The 30-day in-hospital mortality was also evaluated. A multivariate analysis model was created from potentially confusing variables that were statistically significant in an unvaried analysis. *P *< 0.05 was considered statistically significant.

## Results

Two hundred and six patients were included. Most of them were women (54%), with an average age of 62 years. The mean APACHE score was 17 and the mean SOFA score was 5.8. The proportion, according to the RIFLE criteria, for patients at 'risk' was 17%, at 'injury' was 14%, 'failure' was 26% and 'no AKI' was 42%. The relative risk for 30-day in-hospital mortality for the group 'no AKI' was 0.5 (95% CI = 0.39 to 0.63; *P *< 0.001); for the 'risk' group was 1.7 (95% CI = 1.03 to 3.06; *P *= 0.037); for the 'injury' group was 1.66 (95% CI = 0.97 to 2.85; *P *= 0.062); and for the 'failure' group was 2.03 (95% CI = 1.22 to 3.37; *P *= 0.006).

## Conclusions

AKI incidence, according to RIFLE classification, is high in critically ill patients. There is an association between AKI severity and mortality. It is noticeable that patients in the 'risk' group have increased mortality.
